# Impact of prolonged hotel closures during the COVID-19 pandemic on *Legionella* infection risks

**DOI:** 10.3389/fmicb.2023.1136668

**Published:** 2023-02-24

**Authors:** Jhon J. Molina, Magdalena Bennassar, Edwin Palacio, Sebastian Crespi

**Affiliations:** ^1^Environmental Health and Laboratory Services, Biolinea Int., Palma, Spain; ^2^Environmental Analytical Chemistry Laboratory, Department of Chemistry, University of the Balearic Islands, Palma, Spain

**Keywords:** *Legionella*, COVID-19, hotel facility, prolonged closures, domestic water distribution systems

## Abstract

In general, it is accepted that water stagnation and lack or poor maintenance in buildings are risk factors for *Legionella* growth. Then, in theory, the prolonged hotel closures due to the COVID-19 pandemic may have increased the risk of *Legionella* infections. However, there are very few field studies comparing the level of *Legionella* colonization in buildings before the pandemic and the new situation created after the lockdown. The objective of this study was to analyze these differences in a group of hotels that experienced prolonged closures in 2020 due to the COVID-19 pandemic. We have studied the *Legionella* spp. results, analyzed by standard culture, from the domestic water distribution systems of 73 hotels that experienced closures (from 1 to >4 months) during 2020, immediately after the reopening. The results were compared with those obtained in similar samplings of 2019. For the comparative analysis, we divided the hotels in two groups: Group A that have suffered closures for ≤3 months and Group B that remained closed for more than 3 months, both in relation to the opening period of 2019. In the Group B (36 sites), the frequency of positive samples in the hot water system increased from 6.7% in 2019 to 14.0% in 2020 (*p* < 0.05). In the Group A (37 sites), no significant differences were observed. No statistically significant differences were observed in terms of positive sites (defined as hotels with at least 1 positive sample), *Legionella* spp. concentrations and prevalence of *Legionella pneumophila* sg1 between the samplings of the two periods studied. The results suggest that hotels that suffered the longest prolonged closures (> 3 months) could have carried a higher risk of exposure to *Legionella* in the domestic hot water system. These findings highlight the importance of adequate preopening cleaning and disinfection procedures for hotel water systems, and the convenience of considering the most effective disinfection methods especially for hot water systems and after prolonged closure periods.

## 1. Introduction

It is widely accepted that water stagnation in buildings is a risk factor for microbial growth, including opportunistic pathogens as *Legionella* spp. (World Health Organization, 2007; Nisar et al., 2020; Proctor et al., 2020). Among the causes that can favor this microbial proliferation, the following have been cited: decay or decreased disinfectant residual (Boccelli et al., 2003; Barbeau et al., 2005; Goyal and Patel, 2015; Branz et al., 2017; Xu et al., 2018; Huang et al., 2020), accumulation of nutrients (iron and other metals) by means of increase of corrosion (Rakić and Štambuk-Giljanović, 2016; Van der Lugt et al., 2017; Cullom et al., 2020; Martin et al., 2020) or due to leaching from the pipes and scaling (Lytle and Schock, 2000; Nescerecka et al., 2014; Cullom et al., 2020; Li et al., 2020) and development of microbial communities that result in biofilm instability and proliferation of opportunistic microorganisms (Guerrieri et al., 2008; Inkinen et al., 2014; Tsagkari and Sloan, 2018; Rahmatika et al., 2022). Following this body of evidence, current *Legionella* guidelines and regulations recognize the risks associated with stagnation emphasizing the need to avoid dead ends and dead legs, the regular flushing of low-used pipes or the cleaning and disinfection of the water systems after periods of non-use, among others (Health and Safety Executive, 2013; ESGLI, 2017; Ministerio de Sanidad, 2022).

However, the undesired effects of stagnation are highly dependent of different factors including water temperature, flow profiles and total stagnation time (Zhang et al., 2021; Rhoads et al., 2022). It has been this last variable that, in the context of the COVID-19 pandemic, has led many public health institutions and other relevant authorities to promote preventive guidelines for the safe reopening of buildings after months of prolonged mandatory closures. Hotels and other commercial accommodation sites have been severely affected by “stay-at-home” orders and travel restrictions, in particular in touristic destinations where hotels are used mainly for recreational purposes and were not considered essential business. This has been the case in Spain, where the “state-of-alarm” decree (Ministerio de la Presidencia, Relaciones con las Cortes y Memoria Democrática, 2020) forced many hotels to close from March 14, 2020, until the end of June 2020. In addition, many hotels continued to be closed for several more months, due to a lack of customers. This extended closure, even for seasonal hotels that usually close for several months each year during the winter season, is unprecedented, adding an additional period of total closure, during the summer season. In addition, it should be added that the prolonged closure of nonessential buildings also significantly altered the patterns of water demand in the municipal distribution networks of many tourist areas, contributing to further increase the period of stagnation and thus the water age.

In addition, hotels and commercial accommodation sites have been historically associated with a significant number of *Legionella* infections. In Europe, around 15–20% of all reported *Legionella* infections are travel associated (Beauté, 2017). Moreover, it is known that the prevalence of *Legionella* spp. in the water systems of hotels is usually higher than 50%, at least in the Mediterranean area (Borella et al., 2005; Erdogan and Arslan, 2007; Kyritsi et al., 2018; Doménech-Sánchez et al., 2022). Therefore, it is of interest to study the impact that this prolonged closure could have had on the presence of *Legionella* in the domestic water systems of hotels, in particular immediately after the reopening, when, in theory, the consequences of the stagnation could have been more noticeable.

The purpose of this study is to analyze the quality of potable water in reopened hotel establishments, after the prolonged closure due to the COVID-19 pandemic, in terms of the presence of *Legionella* spp. in a group of hotels in the Balearic Islands (Spain), comparing the results with those obtained the previous year in similar samplings. In Spain, hotels are legally obliged to carry out a cleaning and disinfection procedure of the entire domestic water system before reopening the establishment after a period of 1 month of closure, in accordance with an established protocol (Ministerio de Sanidad y Consumo, 2003) and to carry out a sampling for *Legionella* spp. testing, following a standardized procedure (UNE 100030:2017. *Guidelines for prevention and control of proliferation and spread of Legionella in facilities*). To our knowledge, this is the first study of its kind that includes a relatively large number of hotels whose water systems had previously been disinfected through protocolized procedures and compares the results of similar samplings carried out before and after the extended closures due to the COVID-19 pandemic.

## 2. Materials and methods

### 2.1. Experimental design

Water samples collected from a group of hotel facilities in Spain, that had experienced prolonged closures throughout 2020 due to COVID-19 pandemic, were tested for *Legionella* spp. immediately after opening (first sampling). The frequency of positive samples, the prevalence of positive sites, the concentrations of *Legionella* spp. and the presence of *Legionella pneumophila* sg1 were analyzed. The results were compared with those obtained for the same hotels in similar samplings of 2019. All the hotels included in the study operated seasonally. All hotels had their domestic water systems disinfected prior to reopening according to local regulations. All the samplings were conducted at least 2 weeks after the disinfection procedure. For the comparative analysis, the hotels were divided in two groups: Group A that have suffered closures for ≤3 months and Group B that have remained closed for more than 3 months, both in relation to the opening period of 2019.

### 2.2. Definitions

(a) Positive sample: any sample with a positive result of ≥10 CFU/L.
(b) Positive site: hotel with at least 1 positive sample for *Legionella* spp.
(c) Prolonged closure: any closure in a determined hotel of at least 1 month (30 days) in 2020 due to the COVID-19 pandemic, in relation to the normal opening period in 2019; period of prolonged closure calculated as: closing months in 2020 minus closing months in 2019.

### 2.3. Sampling

The samplings were conducted in accordance with the Spanish standard UNE 100030:2017 (*Guidelines for prevention and control of proliferation and spread of Legionella in facilities*). The number of samples collected from each hotel depended on the number of rooms being approximately 0.5 √n for the hot water systems and 0.25 √n for the cold water systems [n = number of rooms; Asociación Española de Normalización (AENOR), 2017]. The sampling points included the cold and hot water tanks and several terminal points, taps and showers, from different areas of the building. All the samples were taken after 1 min of flushing, followed by measuring the temperature and the biocide level (in cold water) before collecting 1 liter of water in a sterile container with sodium thiosulphate pentahydrate (0.01%, w/v) and transported immediately at ambient temperature into an isothermal bag to the laboratory. *Legionella* samples were taken and tested by the same accredited laboratory (Biolinea Int. S.L.). Water temperature was tested using a calibrated digital thermometer at the time of the sample collection after 1 min of flushing.

### 2.4. Detection and enumeration of *Legionella* spp. by culture

The samples were processed within 24 h of collection. The detection and enumeration of *Legionella* spp. in the water samples was carried out according to the UNE-EN ISO 11731:2017 standard. The isolates were further identified and serotyped (*L. pneumophila* serogroup 1, *L. pneumophila* serogroup 2–14 and *Legionella* non-pneumophila species) using latex agglutination reagents (Oxoid, Spain).

Water samples where *Legionella* spp. was not detected or when containing <10 CFU/L were considered negative, and the result equated to 0 CFU/L for the statistical analysis.

### 2.5. Statistical analysis

For the statistical analysis, the 850 data were tested for normality using the Kolmogorov–Smirnov test. The nonparametric chi-squared test was used to compare the proportion of positive results in two groups. The Mann–Whitney U test was used to compare the concentration of *Legionella* spp. IBM SPSS Statistics V22.0 was used for statistical analysis. Results with *p* < 0.05 were considered statistically significant.

## 3. Results

### 3.1. Characterization of the hotel groups

Seventy three hotels were included in the study, 37 in the Group A and 36 in the Group B. The average number of floors and rooms were, respectively, 4.1 ± 2.3 and 177 ± 138 in group A and 5.3 ± 2.9 and 194 ± 125 in Group B. The prolonged closure periods in 2020 for both groups, the number of hotels for each prolonged closure period and the average water temperatures of the samples from the cold and hot water systems from both years are shown in Table 1.

**Table 1:** Prolonged closure periods in 2020 of Groups A and B and average water temperatures of the samples taken in 2019 and 2020 (CWS = cold water system; HWS = hot water system).

	Months of prolonged closure	Number of hotels	Average water temperatures (°C)			
			2019		2020	
			CWS	HWS	CWS	HWS
Group A	≥1–2	8	22.8 ± 1.3	52.7 ± 4.6	25.7 ± 1.8	52.6 ± 4.4
	>2–3	29	21.4 ± 1.3	53.4 ± 6.2	24.7 ± 1.9	51.2 ± 8.4
Group B	>3–4	30	20.7 ± 2.0	56.0 ± 4.7	25.6 ± 2.6	55.9 ± 5.4
	>4	6	19.4 ± 1.5	56.0 ± 5.3	25.9 ± 1.9	56.4 ± 5.5

### 3.2. Microbiological results

Four hundred fourteen water samples were collected in 2019 and 436 in 2020. The proportion of positive samples in the cold water system and the hot water system per year and per group, and the pertinent *p* values is shown in Table 2. Overall, there were 35 positive samples (8.5%) in 2019 and 46 positive samples (10.6%) in 2020 (*p* > 0.05). Only the proportion of positive samples in the hot water system of Group B experienced a statistically significant increase (6.7% in 2019 and 14% in 2020, *p* < 0.05).

**Table 2:** Number of samples (N), number of positive samples (*n*), proportion of positive samples (% pos) per Group (A, B) and year (2019, 2020), and *p* values (CWS = Cold Water System; HWS = Hot Water System).

	Group A			Group B			CWS	HWS	TOTAL	CWS	HWS	TOTAL	*N*/*n* (% pos)	*N*/*n* (% pos)	*N*/*n* (% pos)	*N*/*n* (% pos)	*N*/*n* (% pos)	*N*/*n* (% pos)
2019	55/2 (3.6)	135/21 (15.6)	190/23 (19.2)	75/2 (2.7)	149/10 (6.7)	224/12 (9.4)
2020	59/5 (8.5)	42/19 (13.4)	201/24 (21.9)	85/1 (1.2)	150/21 (14.0)	235/22 (15.2)
*p*	0.102	0.172	0.30	0.110	0.038	0.302

The number of positive sites was 27 (37.0%) in 2019 and 22 (30.1%) in 2020 (*p* > 0.05).

The number of positive samples to *Legionella pneumophila* sg1 was 10 (28.5%) in 2019, involving 9 hotels, and 17 (36.9%) in 2020, from 10 hotels (*p* > 0.05). No statistically significant differences were observed either by groups or by water system.

**Figure 1:**
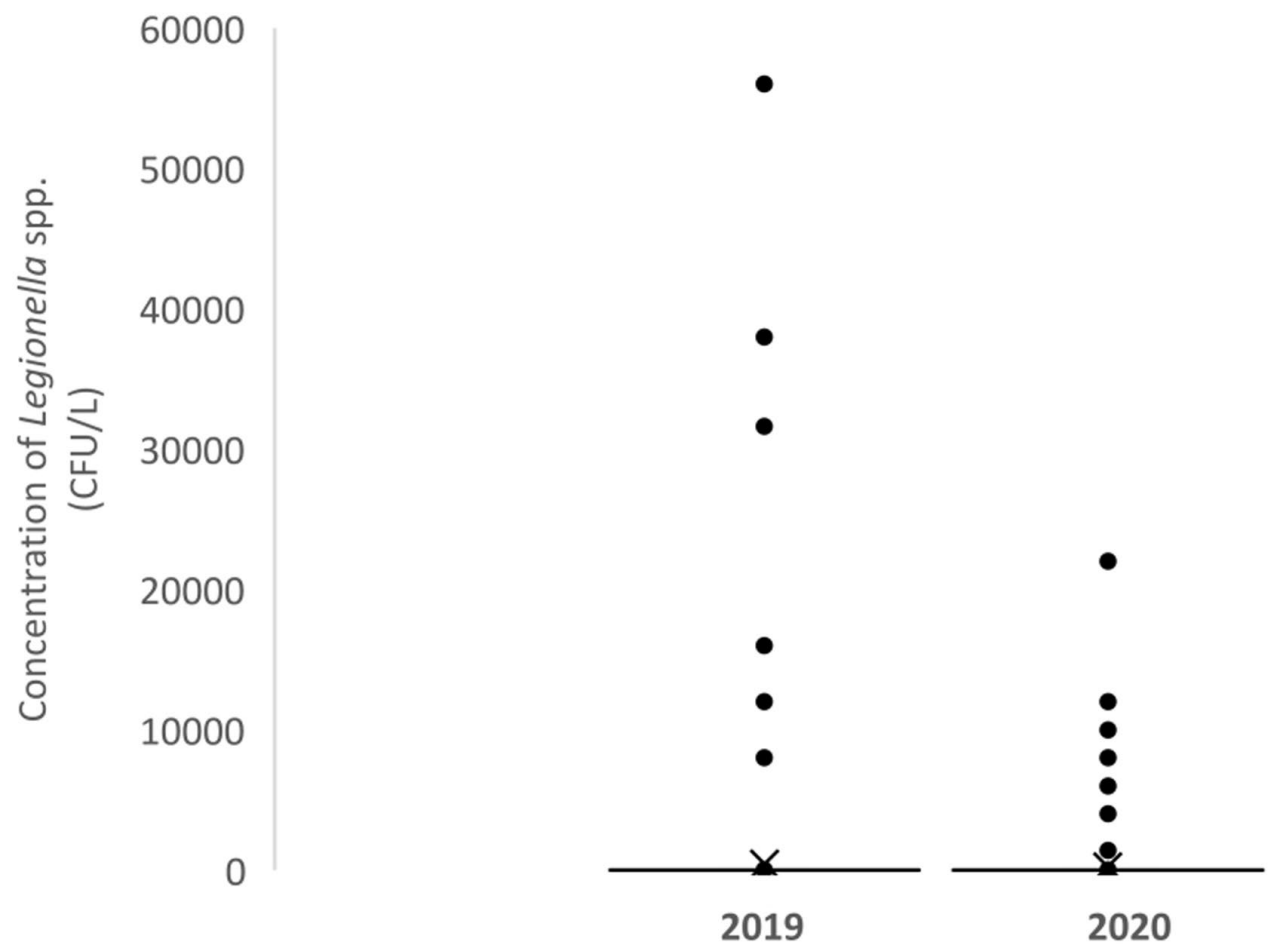
Box plot of *Legionella* spp. concentrations (CFU/L) in 2019 and 2020.

The results of *Legionella* concentrations in the periods 2019 and 2020 for the different samplings are summarized in Table 3. There were no significant differences between the concentrations of *Legionella* spp. found in 2019 and 2020 (Figure 1). No statistically significant differences were observed either by groups or by water system.

**Table 3:** Range of *Legionella* spp. concentrations by water system (cold and hot water systems), year (2019, 2020), and Group (A, B; *n* = number of samples, % = proportion of positive or negative samples, CFU/L = Colony Forming Unit per Liter).

*Legionella* spp. (CFU/L)	Group A			Group B		
	*n*	%	Mean (CFU/L)	*n*	%	Mean (CFU/L)
Cold water system						
2019						
≤1 × 10^2^	1	1.8	8.0 × 10^1^	1	1.3	3.6 × 10^1^
1 × 10^2^–1 × 10^3^	0	0	-	1	1.3	3.6 × 10^2^
≥1 × 10^3^	1	1.8	8.0 × 10^3^	0	0	-
Positive	2	3.6	-	2	2.6	-
Negative	53	96.4	-	73	97.3	-
2020						
≤1 × 10^2^	2	3.4	6.0 × 10^1^	1	1.2	6.0 × 10^1^
1 × 10^2^–1 × 10^3^	2	3.4	1.2 × 10^2^	0	0	-
≥1 × 10^3^	1	1.7	1.0 × 10^4^	0	0	-
Positive	5	8.5	-	1	1.2	-
Negative	54	91.5	-	84	98.8	-
Hot water system						
2019						
≤1 × 10^2^	10	7.4	2.1 × 10^1^	6	4.0	1.5 × 10^1^
1 × 10^2^–1 × 10^3^	6	4.4	1.9 × 10^2^	0	0	-
≥1 × 10^3^	5	3.7	2.4 × 10^4^	4	2.7	1.6 × 10^4^
Positive	21	15.6	-	10	6.7	-
Negative	114	84.4	-	139	93.3	-
2020						
≤1 × 10^2^	8	5.6	2.2 × 10^1^	10	6.7	3.1 × 10^1^
1 × 10^2^–1 × 10^3^	5	3.5	2.4 × 10^2^	4	2.7	4.2 × 10^2^
≥1 × 10^3^	6	4.2	4.5 × 10^3^	7	4.7	9.9 × 10^3^
Positive	19	13.4	-	21	14.0	-
Negative	123	86.6	-	129	86.0	-

## 4. Discussion

Although water stagnation in large buildings, like hotels, is considered a risk factor for microbial proliferation, its real impact in terms of both *Legionella* colonization and *Legionella* infections is not well understood. This is due, in part, to the intrinsic difficulties of studying the effects of building stagnation under real conditions, in numerous buildings, under relatively well controlled conditions and over long periods of time. Thus, there are studies that have analyzed these effects along short periods, e.g., days or weeks (Lautenschlager et al., 2010; Montagnino et al., 2022), studies that have evaluated some buildings (De Giglio et al., 2020; Liang et al., 2021), other studies mixing different types of buildings and water systems (Turetgen, 2021) and, finally, laboratory investigations using lab-scale models (Wang et al., 2012; Rhoads et al., 2017; Tsagkari and Sloan, 2018; Martin et al., 2020). However, field studies carried out with many buildings that have suffered stagnation for relatively long periods and that have been sampled under similar conditions before and after the period of stagnation are rare or non-existent. The COVID-19 pandemic has provided an unprecedented favorable situation to carry out this type of research, by causing prolonged closures of numerous buildings and situations of low demand for water in large areas of the distribution network.

Hotels are ideal buildings for this type of studies. First, because stays in hotels and other commercial tourist accommodation establishments have been associated historically with a very significant number of cases of legionellosis (Beauté et al., 2012) and there is, in Europe, a well-established epidemiological surveillance system, the European Legionnaires’ disease Surveillance Network (ELDSNet), for cases of legionellosis associated with travel (European Centre for Disease Prevention and Control, 2017). Second, because the relatively high rates of *Legionella* colonization in hotels makes it relatively easy to obtain meaningful statistical data when conducting pre-and post-intervention studies. And finally, because it is a sector that, at least in Spain, is regulated in terms of *Legionella* prevention thus following standardized guidelines for certain practices, such as annual disinfection procedures and routine samplings. We have taken advantage of this situation by analyzing the results of a group of hotels for which we had microbiological data for the year 2019, corresponding to a first sampling carried out after the annual disinfection, which, in Spain, is mandatory and must be carried out within 1 month before the reopening in seasonal hotels. This, together with the fact that the sampling must be done in a standardized way in terms of the number of samples and the sampling points, has allowed us to make a comparative analysis of the pre-and post-pandemic results, ensuring a reasonable degree of homogeneity in both initial yearly samplings.

The results of this study suggest that only hotels that suffered the longest prolonged closures (>3 months) could have carried a higher risk of exposure to *Legionella* in the domestic hot water system. Indeed, only the Group B showed a significant increase in the proportion of positive samples for *Legionella* spp. in 2020, after their reopening. In contrast, the hotels in Group A, with closures ≤3 months, although they tended to have higher frequencies of positives, these were not statistically significant. However, these data must be balanced against the absence of significant changes in the number of positive hotels, *Legionella* concentrations and presence of *Legionella pneumophila* sg1. It is very likely that these factors, especially the last two, play an important causal role in the chain of infection, given the preponderance of the *Legionella pneumophila* sg1 in the set of *Legionella* infections (Yu et al., 2002) and the general relevance of the concentrations of the pathogenic germ at source in quantitative microbial risk assessments (Hamilton et al., 2019; Sharaby et al., 2019). Then, this is a relatively modest impact, being limited only to the frequency of positive samples in the hot water system, which, in the absence of relevant changes in the bacterial loads and prevalence of *Legionella pneumophila* sg1, might have little relevance in terms of actual infections, although we do not have data on this last aspect. In fact, the data on the incidence of legionellosis cases associated with travel for 2020 are hardly relevant given the significant travel restrictions that still existed this year, being difficult to compare with the previous year (the number of reported cases to the travel-associated surveillance scheme of ELDSNet decreased by 67% in 2020 compared with 2019; European Centre for Disease Prevention and Control, 2020).

In general, the results of our study are in line with other studies that have also observed increased risk of exposure to *Legionella* after prolonged facility closures due to the COVID-19 pandemic (Turetgen, 2021), although in our study only the hotels that were closed the longest showed significant increases. This seems logical, considering that the total stagnation time and water age in buildings are relevant factors in the persistence of microbial colonization, resulting in higher bacterial loads (Rhoads et al., 2016) and the formation of more dense and complex biofilms (Tsagkari and Sloan, 2018). We must highlight that our study evaluated the presence of *Legionella* following the reopening of the hotels, after having carried out a mandatory cleaning and disinfection of the water systems, that is, under normal operating conditions, in contrast to other studies that were carried out before returning to normal activities (De Giglio et al., 2020; Liang et al., 2021). In this sense, our results suggest that these cleaning and disinfection procedures, in preparation for the reopening after the closure induced by COVID-19 pandemic, could have been insufficient to eliminate the highest bacterial loads and dense biofilms in the hotels that suffered the longest shutdowns.

The fact that significant increases in the frequency of positive samples have been observed only in the hot water system, and not in the cold water, may be due to several factors. Firstly, different studies suggest that *Legionella* preferentially colonize hot water systems of buildings (Nisar et al., 2020; Doménech-Sánchez et al., 2022; Gamage et al., 2022). Second, because the Spanish regulations allow choosing between chemical or thermal disinfection, it is possible that, at least in a significant number of installations, the hot water system had been thermally disinfected (raising the water temperature up to 70°C for 2 h), a method that is generally considered less effective and has fewer lasting effects than chemical disinfection (Molina et al., 2022). Since we have not investigated the precise methodology used in pre-opening disinfections, we cannot rule out that a preferential use of one or the other may have affected the results. Finally, the presence of biocide in the cold water system (in Spain, 0.2–1.0 mg/L of residual free chlorine is required in cold drinking water) may have helped to recover the baseline conditions in this system earlier after reopening.

Our study has several strengths and some limitations. First, because of the regulations in Spain, we had the opportunity to compare results from the same group of hotels in 2019 and 2020 in quite similar conditions (first sampling after the annual disinfection procedure), thus avoiding possible bias due to longer periods of normal operation after reopening and subsequent recovery of base-line conditions. The average hot water temperatures at which the samples were taken in both years did not differ significantly, thus allowing a fair comparative analysis between the 2 years. Our study also benefits from the fact that all samplings and analysis were done by the same laboratory, which has accredited both procedures, sampling and testing, thus avoiding the possible biases that could occur with different samplings and/or analysis systems in other studies. Finally, the methodology used for the domestic water system disinfection was essentially the same for all the sites, following the same regulation, so, in theory, this procedure should not have affected from 1 year to another.

The study also presents some limitations. First, both the number of hotels and the number of samples is too low for deriving strong conclusions. We also acknowledge that the proportion of positive samples in 2019 for the Group B (6.7%) was quite low in comparison of the proportion of positive samples for Group A (15.6%). The causes of this difference are not clear, given that the structural characteristics of both groups of hotels (number of floors, number of rooms) were not much different, although we cannot completely rule out some significant structural and operational differences (piping materials, flushing regimes, chlorine levels, etc.) that were not considered and could have conditioned the results in one way or another. Indeed, hot water temperatures in Group B were a bit higher than in Group A. However, we know that Group B presented a similar percentage of positives in 2021 in relation to 2019 (7.5%, data not shown in the results section), which suggests that the increase observed in 2020 was genuine and not the result of an exceptionally low result from the previous year. We also acknowledge the limitations of culture methodology for estimating the number of viable bacterial cells in water samples. It is known that stagnation and other environmental stresses can promote generation of viable but non-culturable (VBNC) *Legionella* cells (Li et al., 2014). So, it is likely that our study underestimated the actual bacterial load, especially in the 2020 sampling. Finally, it should be noted that the 2020 samplings were generally carried out in the middle of summer, while in 2019 they were carried out much earlier, mostly in winter. This may have at least affected the results in the cold water system (the average temperature of the cold water samples in 2020 was higher than in 2019). However, this fact does not seem to have had relevant effects since we have not observed significant increases in this system. Likewise, it is possible that this fact affected the pattern of water use in the hotels but in absence of precise data, e.g., occupancy rate, water consumption per person, it is difficult to assess this possible effect.

The results of this study are only applicable to hotels that operate seasonally, which abound in the tourist areas of the Mediterranean basin. It has been suggested that seasonal hotel operation, as opposed to hotels that operate all year around, favors the colonization by legionellae in hotel water systems (Mouchtouri et al., 2007). In this sense, seasonal hotels also could offer an interesting field model to investigate the effect of prolonged stagnation. However, in these cases the stagnation is not usually total since the facilities are subject to a certain degree of both water use and maintenance. On the other hand, the closing periods tend to occur in typically regular periods and generally shorter than those forced by the pandemic. In this case, all the hotels included in the study operated seasonally, but they suffered longer closures than the merely seasonal ones, which also included part of summer. Situations of prolonged closures in hotels can also occur, to a greater or lesser extent, in other cases, for example in periods of low occupancy or during closures for renovations or modifications, although again, in these cases, the stagnation is usually only partial or temporary.

In summary, this study suggests that prolonged closures of hotels longer than 3 months due to the COVID-19 pandemic caused an increased risk of exposure to *Legionella* in the hot water systems. However, the practical consequences of this increase in terms of actual *Legionella* infections, in the absence of other risk factors (higher *Legionella* concentrations, more positive establishments, or more prevalence of *Legionella pneumophila* sg1) may have been relatively modest. In any case, these findings highlight the importance of adequate preopening cleaning and disinfection procedures for hotel water systems after periods of closure and the convenience of considering the most effective methods after closures that are longer than usual, especially in hot water systems.

## Data availability statement

The raw data supporting the conclusions of this article will be made available by the authors, without undue reservation.

## Author contributions

SC: conceptualization. JM and MB: data curation. JM, SC, and MB: investigation. JM and EP: data analysis. SC and EP: supervision. SC and JM: writing—original draft. SC, JM, and EP: writing—review and editing. All authors contributed to the article and approved the submitted version.

## Funding

This work was supported by ATA Ecotecnologia e Higiene del Agua S.L., Palma, Spain. Code: ATA-Bio LEGIONELLA/COVID-19 study.

## Conflict of interest

The authors declare that the research was conducted in the absence of any commercial or financial relationships that could be construed as a potential conflict of interest.

## Publisher’s note

All claims expressed in this article are solely those of the authors and do not necessarily represent those of their affiliated organizations, or those of the publisher, the editors and the reviewers. Any product that may be evaluated in this article, or claim that may be made by its manufacturer, is not guaranteed or endorsed by the publisher.
